# Binge Ethanol Prior to Traumatic Brain Injury Worsens Sensorimotor Functional Recovery in Rats

**DOI:** 10.1371/journal.pone.0120356

**Published:** 2015-03-13

**Authors:** Ian C. Vaagenes, Shih-Yen Tsai, Son T. Ton, Vicki A. Husak, Susan O. McGuire, Timothy E. O’Brien, Gwendolyn L. Kartje

**Affiliations:** 1 Research Service, Edward Hines Jr. VA Hospital, Hines, Illinois, United States of America; 2 Department of Molecular Pharmacology and Therapeutics, Loyola University Medical Center, Maywood, Illinois, United States of America; 3 Neuroscience Research Institute, Loyola University Medical Center, Maywood, Illinois, United States of America; 4 Department of Mathematics and Statistics, Loyola University of Chicago, Chicago, Illinois, United States of America; 5 Department of Anesthesiology, College of Medicine, University of Illinois at Chicago, Chicago, Illinois, United States of America; Uniformed Services University, UNITED STATES

## Abstract

A significant number of patients suffering from traumatic brain injury (TBI) have a high blood alcohol level at the time of injury. Furthermore, drinking alcohol in a binge-like pattern is now recognized as a national problem, leading to a greater likelihood of being injured. Our objective was to determine the consequences of a binge paradigm of alcohol intoxication at the time of TBI on long-term functional outcome using a sensitive test of sensorimotor function. We trained adult, male, Sprague Dawley rats on the skilled forelimb reaching task and then administered a single binge dose of ethanol (2g/kg, i.p.) or saline for three consecutive days (for a total of 3 doses). One hour after the final ethanol dose, rats underwent a TBI to the sensorimotor cortex corresponding to the preferred reaching forelimb. Animals were then tested for seven weeks on the skilled forelimb reaching task to assess the profile of recovery. We found that the group given ethanol prior to TBI displayed a slower recovery curve with a lower recovery plateau as compared to the control group. Therefore, even a relatively short (3 day) episode of binge alcohol exposure can negatively impact long-term recovery from a TBI, underscoring this significant public health problem.

## Introduction

1.7 million Americans suffer a traumatic brain injury (TBI) every year[1]. Of those hospitalized, approximately 50,000 die [1,2], and those who survive face a difficult path to recovery. Many TBIs lead to permanent functional deficits, with approximately 5.3 million Americans in need of lifelong assistance for daily living as a result of a TBI [3].

Given the significance of this health crisis, there has been considerable research attempting to assess the degree to which lifestyle factors may influence the outcome of TBI. One such factor investigated is alcohol use, and in this regard, the “binge” pattern of alcohol consumption is currently on the rise among certain demographics [4]. The National Institute on Alcohol Abuse and Alcoholism (NIAAA) defines alcohol binge as a mode of consumption that leads to a blood alcohol level greater than the legal limit 0.08% (80 mg/dl, ie, 5 drinks for men and 4 drinks for women in two hours) [5]. Binge drinking is a common practice among Americans; a recent study found that approximately 1 out of every 6 adults in America binge drink four times a month [6]. Furthermore, binge alcohol consumption is correlated with increased risky behavior that can lead to injuries, including TBI [7]. Accordingly, it has been estimated that 30–50% of TBI patients have a blood alcohol level above the legal limit at the time of injury [8]. In fact, intoxicated TBI patients entering the emergency room typically have a blood alcohol level much higher than the legal limit at approximately 170mg/dl [9].

The few animal studies of behavioral recovery after binge ethanol combined with TBI have given equivocal results. Depending on the injury severity and the dose of ethanol administered, recovery after TBI may or may not be hindered [10, 11]. In one report, four weeks of binge ethanol treatment followed by ethanol intoxication at the time of TBI worsened some aspects of spatial learning in rats [12]. Earlier work has shown that a high single binge dose of ethanol can worsen recovery of sensorimotor function.[10, 13] However, these deficits may be transient [13]. Therefore, it remains unclear whether binge ethanol given prior to TBI affects long-term functional outcome, especially given that most studies focused on short recovery time points.

Given these findings, we set out to examine whether a repeated binge regimen of ethanol delivered prior to TBI would lead to an impaired *long-term* sensorimotor functional recovery. We used a rodent model of TBI, the controlled cortical impact (CCI), and evaluated the outcome with a sensitive measure of sensorimotor function, the skilled forelimb reaching task. We found that rats treated with a three day regimen of binge ethanol prior to TBI exhibited worse sensorimotor recovery on this skilled task as compared to rats with TBI only.

## Methods

### Ethics Statement

Animal use was approved by the Institutional Animal Care and Use Committee (IACUC) of Edward Hines Veterans Affairs Hospital permit #H13–001.

### Animals

23 Sprague Dawley male rats (Harlan, IN) 10 weeks old and weighing approximately 260grams at the beginning of training, were used in this study. Rats were housed two in a cage in a fully accredited animal care facility with a 12-hour light/dark cycle. Rats were food-restricted to 95% of their predicted weight by age in order to encourage learning the behavioral task. All animals were number coded and investigators were blind to the animal treatment groups.

### Behavioral Testing

**Fig 1:**
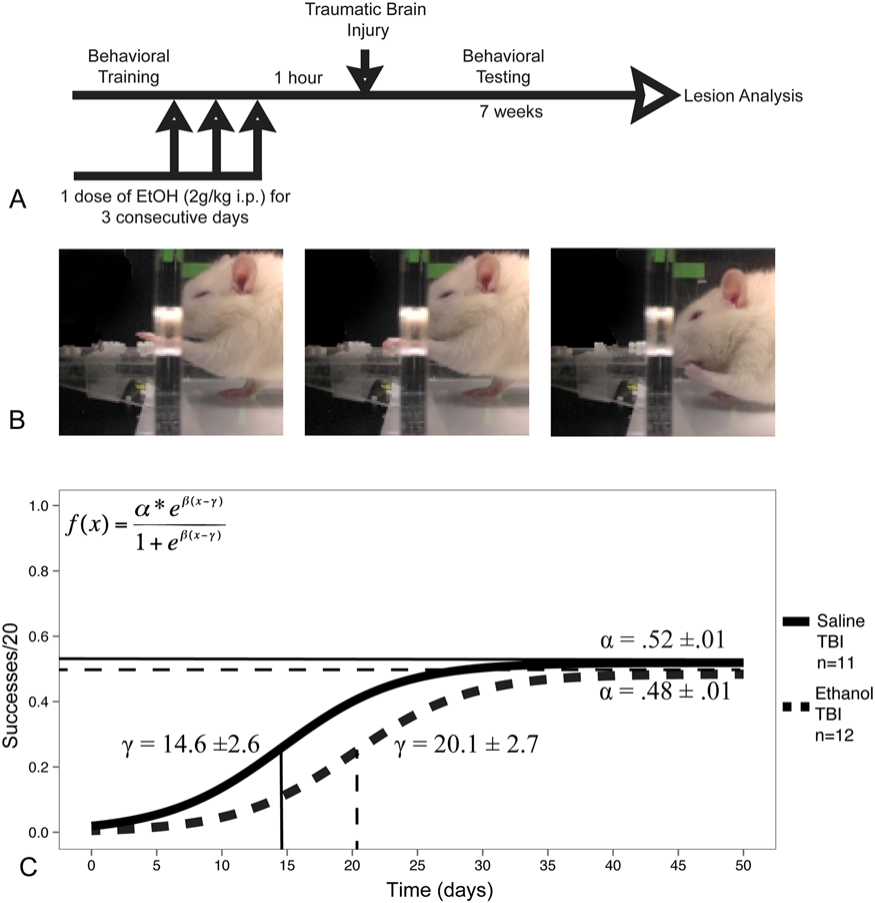
A. Experimental design schematic. B. Representative kinematic sequence of a non-injured rat during the skilled reaching task. C. Fitted curves of the estimated group-level logistic recovery for rats given either three doses of binge ethanol (2g/kg) or vehicle control prior to TBI. The group differences in the midpoint (γ) and asymptote (α) of recovery were found to be statistically significant (p<.05).

To examine complex sensorimotor forelimb function, the skilled forelimb reaching test was used (Fig. 1B), as previously designed [14]. Briefly, rats were weighed and placed in a Plexiglas chamber (30x36x30 cm) with a small rectangular opening (1.5 x 3 cm) on one wall with an external shelf underneath the opening. Rats were trained to reach through the opening for pellets placed on the shelf. A single trial consisted of 20 sugar pellets (45 mg; BioServ, Flemington, NJ) placed successively on the shelf. A successful reach was defined as one in which the animal, in one attempt, reached through the opening, grasped the pellet, and brought it back to its mouth. During the course of training, limb preference was first determined for each animal. Rats were trained to a baseline success rate of 14 out of 20 successful reaches using the preferred forelimb. Once baseline scores were achieved, the animal received a TBI to the sensorimotor cortical area corresponding to the preferred forelimb, resulting in impairment on this task. Animals were tested Mon-Fri for seven weeks post-TBI and sessions were video recorded for further analysis.

### Alcohol Administration

Following behavioral training and prior to TBI, rats in the ethanol treatment group (n = 12) were administered 3 consecutive doses of ethanol (2g/kg, i.p.) once per day for 3 days. Control animals (n = 11) received an equal volume of 0.9% saline, given using an identical dosing schedule. This ethanol dose leads to blood alcohol levels typical of a TBI patient entering the emergency room (~170 mg/dl) (Bombardier and Thurber, 1998) and is well tolerated in rats.

### Blood Alcohol Concentration Quantification

100μl of blood was drawn from the tail vein of each rat one hour after the last dose was given, and the blood alcohol concentration (BAC) was quantified using an oxygen-rate alcohol analyzer (Analox Instruments, London, UK).

### TBI model-Controlled Cortical Impact (CCI)

All rats were anesthetized with 3% isoflurane delivered as an inhalant with 5% oxygen. During surgery, animal body temperature was maintained at 38°C using the physitemp TCAT-2DF Controller system (Physitemp Instruments Inc, Clifton, NJ). Rats were placed in a stereotaxic instrument, followed by a small scalp incision. Using a trephine, a 5mm diameter disc of skull was removed to expose the primary forelimb motor cortex (1.5mm anterior, 2.5 mm lateral from bregma, [15]) of the hemisphere opposite the preferred reaching forelimb. The CCI was delivered using a double acting electromagnetic piston mounted on a stereotaxic crossbar, angled for cortical impact (diameter: 3mm, velocity: 2.5m/sec, depth: 2mm, dwell time: 250 msec)(Impact One, MyNeurolab, St. Louis, MO). The CCI model results in a reproducible lesion, typified by both focal and diffuse brain damage. Following impact, the disk of bone was put back in place and the scalp incision closed with sutures. Animals were returned to their home cages.

### Lesion Analysis

After completion of behavioral testing, rats were overdosed with sodium pentobarbital (100 mg/kg, i.p.) and transcardially perfused with 0.9% saline followed by 4% paraformaldehyde. Brains were extracted and cryosectioned at 40 microns and stained for Nissl. To determine the lesion size, every 12^th^ section between 2.7mm anterior and -1.7 mm posterior to bregma was scanned using a flatbed scanner and traced in Adobe Photoshop to provide an area. The percentage of hemisphere damage was computed using the following formula: $(\frac{\sum_{}uninjuredhemisphere-\sum injurehemisphere}{\sum uninjuredhemisphere})x100$

This method has been used extensively by us [16–19] and others [20–22].

### Statistical analysis

All data analysis was performed using either SAS (SAS Institute, Cary, NC, USA) or R: *A Language and Environment for Statistical Computing*. Skilled reaching data was fit using a nonlinear, 3-parameter, binomial logistic, mixed effects model:$\frac{\alpha e^{\beta (x-\gamma )}}{1+e^{\beta (x-\gamma )}}$. α is the horizontal asymptote as x → ∞, β is the slope, and γ is the x value at which the response is α/2. α and β were modeled by fixed effects and allowed to vary by group while γ was modeled by random effects. Thus, our model constitutes a nonlinear mixed model approach, which has been shown by us and others to exhibit greater sensitivity to real effects and directly models parameters of interest including final recovery and rate of recovery [17, 23, 24]. Group parameters were compared using a likelihood based chi squared test with an alpha level of 0.05. Lesion volume comparisons were done using an unpaired t-test, with α = .05 as the cutoff for statistical significance.

## Results

### Repeated Binge Ethanol and Sensorimotor Recovery

After TBI, all animals in both groups showed a significant deficit in performance on the skilled forelimb reaching task. Furthermore, over the course of seven weeks of testing, all animals showed some increase in spontaneous sensorimotor recovery (Fig. 1C), and the difference in the estimated slopes of recovery (β) were not statistically significant between the saline and ethanol treated groups. However, the midpoint (γ) and the recovery asymptote (α) were found to be significantly different, with ethanol treated rats reaching the midpoint of recovery at a later time point (20.1±2.6 days compared to 14.61±2.7 days in saline treated rats, p<0.05). The asymptote of recovery was also found to be lower in the ethanol treated group (0.48 ± 0.01 compared to 0.52 ± 0.01 in saline treated rats, p<0.05). Thus, binge ethanol treated rats took significantly longer to reach their recovery plateau, and their final level of recovery was less than that of saline treated rats.

### Lesion Analysis

**Fig 2:**
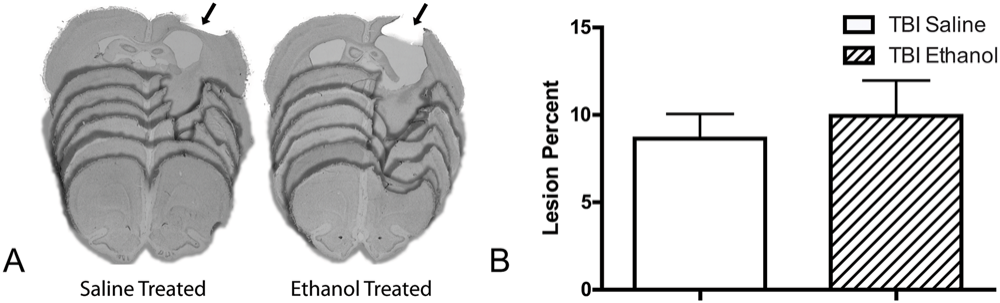
A. Representative nissl stained coronal sections of saline treated and ethanol treated seven weeks post traumatic brain injury. The arrows indicate the location of the lesion. B. Lesion size expressed as a percent of the unlesioned hemisphere. No significant difference was found between saline and ethanol treated animals p>.05 (unpaired t-test). Error bars denote the ± SEM.

TBI lesion size was quantified at the end of the behavioral testing (Fig. 2) and taken together with the exhibited behavioral deficits indicated a mild to moderate TBI in all animals. No statistically significant difference was found between groups (9.9% ± 2.0 in ethanol treated rats vs. 8.7% ± 1.4 in saline treated rats, unpaired t-test, p>.05).

### Blood Alcohol Concentration

Blood ethanol concentrations were 185 ±20.7 mg/dl (mean ± SEM) at 1 hour after i.p. injection (the time of the TBI).

### Rat Weight

The weights of alcohol and saline treated animals were not significantly different on the day of injury (349.6 ± 10.98g in ethanol treated rats vs. 347.8 ± 13.3g in saline treated rats, mean ±SEM, unpaired t-test, p>.05) or 24 hours later (343.0 ± 10.17g in ethanol treated rats vs. 343.4 ± 10.2g in saline treated rats, mean ± SEM, unpaired t-test, p>.05). Furthermore, we did not find a statistically significant difference in the number of days until each animal reached their respective pre-injury baseline weight after injury (5.5 ± 1.4 days in ethanol treated rats vs. 4.7 ± 1.3 days in saline treated rats, unpaired t-test, p>.05).

## Discussion

Our results show that ethanol administered in a repeated binge fashion (2g/kg, i.p.) over three days before a mild to moderate TBI primarily involving the forelimb motor cortex led to a significantly slower recovery and a worse overall performance on a test of skilled forelimb function.

To our knowledge, this is the first study to demonstrate that binge ethanol given prior to a TBI and at the time of injury leads to long-term deficits on a sensorimotor task with less spontaneous recovery when compared to rats with no ethanol and the same TBI. The limited previous work evaluating the effects of pre-TBI ethanol on sensorimotor recovery has given equivocal results. For example, research using the horizontal beam walk task found that rats given a single dose of 1.5g/kg, i.p. or 2.5g/kg, i.p. ethanol delivered 40 minutes prior to a TBI (CCI) exhibited a faster recovery over the first post-TBI week than animals given saline or 3 g/kg ethanol i.p., indicating a neuroprotective effect of those lower doses of ethanol [10]. We did not find a similar effect when assessing recovery during the first two weeks post injury. In our study, even in the first weeks post-injury, binge ethanol treated rats exhibited a trend towards poorer performance when compared to the control group. Importantly, we used a similar dose to Kelly et al but with a three day dosing schedule. Therefore, the repeated binge regimen as done in our study may have been the reason for the lack of neuroprotection and the overall worse outcome. Another study using a chronic binge model (6 weeks of 35% ethanol derived calories, liquid diet) followed by a lateral fluid percussion (LFP) TBI, also showed no significant difference on beam walk task performance when compared to pair-fed controls [25]. However, examination of their injury parameters showed a location in the lateral and posterior aspects of the parietal and temporal cortex, a different location as compared to the neocortical sensorimotor location of the lesions in our study. Furthermore, the beam walk task is a different type of behavioral assessment as compared to the skilled reaching task, which may be more sensitive to recovery [26]. Our study is in support of a previous report using a battery of neurologic tests to evaluate sensorimotor outcome. Rats given a single dose of ethanol (3g/kg per gavage) prior to a severe TBI (LFP) displayed a worse neuroscore 24 hours after injury [13]. Taking these studies together, one interpretation is that binge ethanol impairs sensorimotor recovery when the binge is repeated (3 days as in our study) or when the TBI is severe in the context of a single binge dose of ethanol. Our work is continuing to investigate this important question of lesion location and severity in regard to neurological outcome when under the influence of binge ethanol.

Given the above findings, how might binge ethanol worsen sensorimotor recovery without an apparent effect on lesion volume? It has been shown that ethanol modulates a number of processes involved in CNS recovery, including adrenergic, purinergic [27] and glutamatergic signaling (for a review see [28]). More recent work has demonstrated that even without a TBI a single binge ethanol exposure is enough to induce brain damage as measured by glial reactivity and neurodegeneration [29]. Interestingly, another mechanism by which binge ethanol may worsen recovery is through effects on neural stem cells. It has been suggested that neural precursor cells integrate into the dentate gyrus to enhance functional recovery [30]. Pertinent to our work, studies have demonstrated that ethanol may suppress neurogenesis, thereby repressing this mode of post-injury compensation [31]. Our current studies are aimed at determining the mechanism of how binge ethanol affects neural precursor cells in the setting of TBI.

How our results in this study translate to the human condition is still unclear. Among the many variables that make our rodent model different from humans is the route of administration of ethanol, where human consumption is by the oral route. However, this route can result in a wider range of blood levels as compared to the i.p. route primarily due to variables in absorption and metabolism. Therefore, we chose to begin our studies using the i.p. route of administration to achieve the most consistent blood alcohol levels as possible in all rats at the time of TBI in order to give the clearest interpretation of our behavioral results. In our current studies we have added the oral gavage route to better emulate the human mode of ethanol consumption.

In conclusion, this study demonstrates that even a short (3 day) repeated binge ethanol exposure prior to a TBI leads to a slower and worse overall outcome on a skilled task of forelimb function in young adult rats. If these results are translatable to humans, given that the *overall* prevalence of binge drinking among American adults is estimated at 17% (even higher among younger demographic groups) and that binge drinkers have a greater risk of TBI, a slowed and reduced recovery represents a significant public health concern [6, 32].

## Supporting Information

S1 Dataset Rodent behavioral and physiological data. (XLSX)
