# Correction: Binge Ethanol Prior to Traumatic Brain Injury Worsens Sensorimotor Functional Recovery in Rats

**DOI:** 10.1371/journal.pone.0149793

**Published:** 2016-02-12

**Authors:** Ian C. Vaagenes, Shih-Yen Tsai, Son T. Ton, Vicki A. Husak, Susan O. McGuire, Timothy E. O’Brien, Gwendolyn L. Kartje

In the Funding section, the grant number from the funder National Institute on Alcohol Abuse and Alcoholism is listed incorrectly. The correct grant number is: T32 AA013527.

## References

[1] Vaagenes IC, Tsai S-Y, Ton ST, Husak VA, McGuire SO, O’Brien TE, et al (2015) Binge Ethanol Prior to Traumatic Brain Injury Worsens Sensorimotor Functional Recovery in Rats. PLoS ONE 10(3): e0120356 doi: 10.1371/journal.pone.0120356 25768795 10.1371/journal.pone.0120356 PMC4359156

